# T‐cell lymphoma, B‐cell lymphoma, and myelodysplastic syndrome harboring common mutations: Trilineage tumorigenesis from a common founder clone

**DOI:** 10.1002/jha2.354

**Published:** 2021-12-03

**Authors:** Kyoko Yoshihara, Yasuhito Nannya, Ikuo Matsuda, Mami Samori, Nobuto Utsunomiya, Masaya Okada, Seiichi Hirota, Seishi Ogawa, Satoshi Yoshihara

**Affiliations:** ^1^ Department of Hematology Hyogo College of Medicine Hospital Nishinomiya Japan; ^2^ Department of Pathology and Tumor Biology Kyoto University Kyoto Japan; ^3^ Department of Surgical Pathology Hyogo College of Medicine Nishinomiya Japan; ^4^ Department of Transfusion Medicine and Cellular Therapy Hyogo College of Medicine Hospital Nishinomiya Japan

**Keywords:** angioimmunoblastic T‐cell lymphoma, common mutations, diffuse large B‐cell lymphoma, founder clone, multilineage tumorigenesis, myelodysplastic syndrome

## Abstract

A 64‐year‐old man with angioimmunoblastic T‐cell lymphoma (AITL) subsequently developed diffuse large B‐cell lymphoma (DLBCL) and myelodysplastic syndrome (MDS). Genomic profiling of AITL, DLBCL, and MDS samples revealed that the tumor cells from all samples shared common mutations in *TET2* and *DNMT3A*. In addition, the *IDH2* mutation was observed in AITL, and *TP53* mutation was observed in DLBCL and MDS. These findings illustrate the clonal relationship between AITL and DLBCL in addition to AITL and MDS, with the latter being increasingly reported. The present findings strongly support the theory of multistep and multilineage tumorigenesis from a common founder clone.

## 1 INTRODUCTION

Recent studies have suggested that tumorigenesis in some hematologic malignancies is initiated by genetic events in hematopoietic stem cells or precursor cells (founder mutations), followed by additional events which are associated with the lineage specificity of the subsequent malignancies. Angioimmunoblastic T‐cell lymphoma (AITL) has been considered to be a model of this hypothesis of multi‐step lymphoma/leukemogenesis [1, 2].

Here, we present a patient with AITL who subsequently developed diffuse large B‐cell lymphoma (DLBCL) and myelodysplastic syndrome (MDS). The tumor cells of AITL, DLBCL, and MDS in this patient shared common mutations in *TET2* and *DNMT3A*, suggesting that hematologic malignancies in the three lineages evolved from a common founder clone.

## 2 CASE PRESENTATION

**FIGURE 1:**
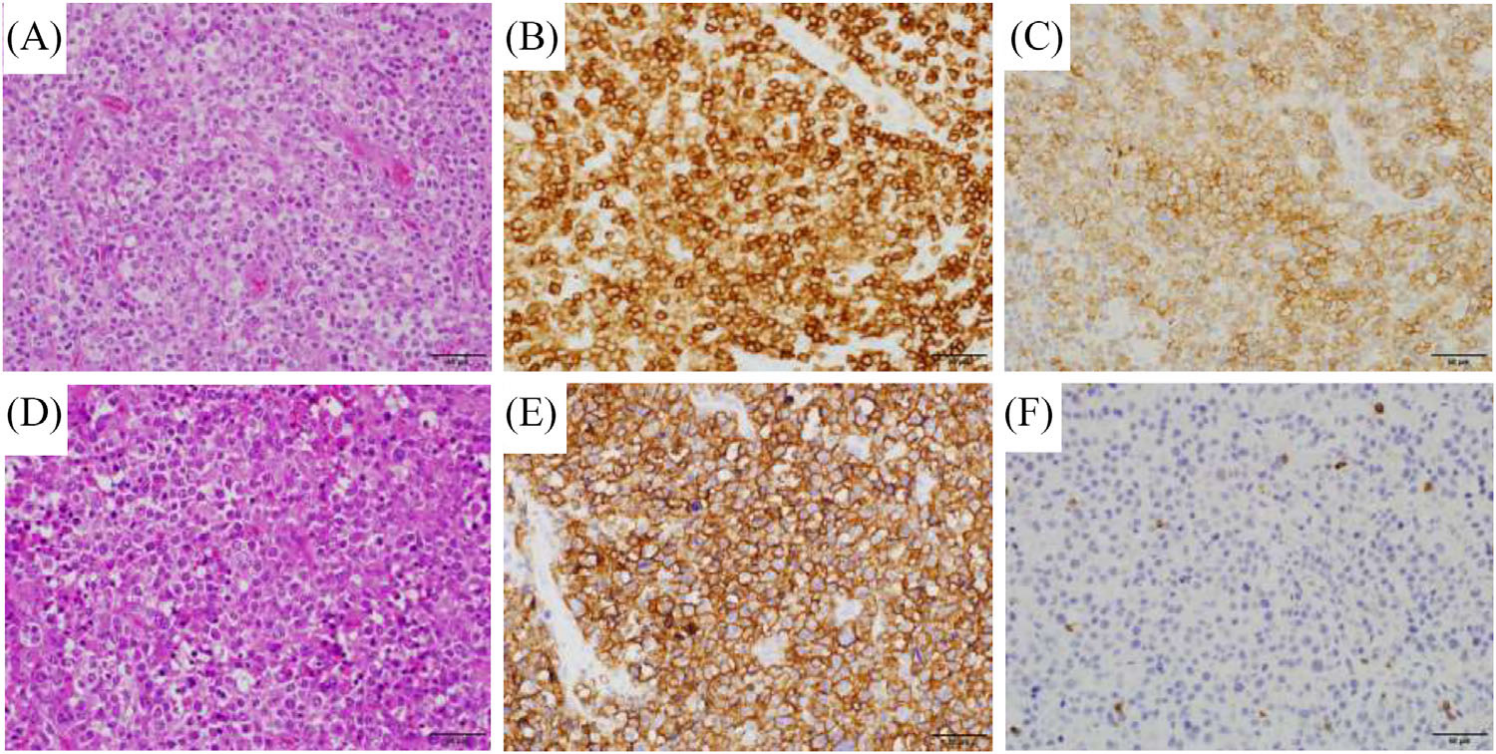
**(A‐C) Histopathological image of angioimmunoblastic T‐cell lymphoma (AITL) of this case at high power view**. (A) Hematoxylin‐eosin (HE) image. Atypical clear cells of medium size infiltrated with expansion of high endothelial venules. (B) The tumor cells were positive for CD3 immunohistochemistry. (C) The tumor cells were positive for PD‐1 immunohistochemistry. **(D‐F) Histopathological image of diffuse large B‐cell lymphoma (DLBCL) of this case at high power view**. (D) HE image. (E) CD20 immunohistochemistry. (F) CD3 immunohistochemistry. For all of the images, bars represent 50 μm

A 64‐year‐old man with systemic lymphadenopathy was diagnosed with AITL (Figure 1A‐C). The patient achieved complete response with six courses of CHOEP chemotherapy (comprising cyclophosphamide, doxorubicin, vincristine, etoposide, and prednisone). He proceeded to upfront autologous stem cell transplantation with the LEED regimen, consisting of high‐dose melphalan, cyclophosphamide, etoposide, and dexamethasone. Thirty‐one months after the diagnosis of AITL (26 months after autologous transplantation), he developed perforation of the terminal ileum. Histopathological analysis of formalin‐fixed paraffin embedded (FFPE) specimens of the surgically resected ileum revealed infiltration of atypical large lymphoid cells which were immunohistochemically positive for CD20, Bcl‐2, and MUM‐1, while being negative for CD3 and CD10 (Figure 1D‐F). In situ hybridization for Epstein‐Barr virus (EBV)‐encoded small RNA was negative. There was no histological evidence of AITL. Diagnosis was established as DLBCL, non‐germinal center B‐cell type. After two courses of R‐CHOP chemotherapy, the bone marrow was examined because of delayed hematopoietic recovery. While dysplasia was not evident morphologically, the cytogenetic analysis revealed a complex karyotype including deletion of 5q, monosomy 7, and trisomy 8, demonstrating the presence of MDS. The bone marrow was reexamined following four courses of R‐GDP chemotherapy, which then showed apparent trilineage dysplasia without an excess of blast. Although he received treatment with azacytidine, there was progressive cytopenia with an increased blast count. The patient died 4 years after the initial diagnosis of AITL due to infections.

**FIGURE 2:**
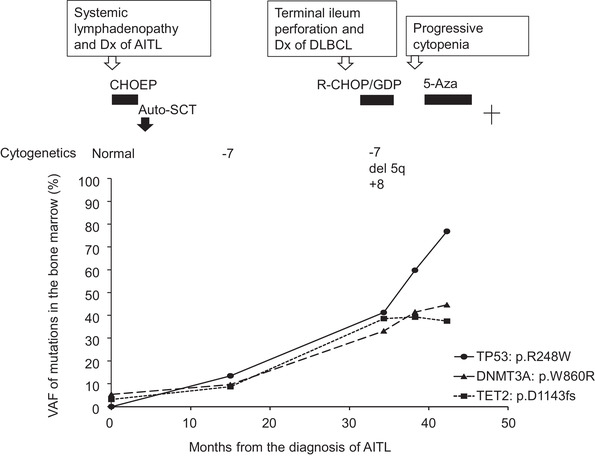
Kinetics of variant allele frequency of the mutations in the bone marrow during the clinical course of the patient

Genomic profiling of AITL, DLBCL, and MDS samples from this patient was performed using targeted sequencing of a panel of genes after obtaining written informed consent. For AITL and DLBCL, DNA was obtained from the FFPE specimen, from which the lymphoma‐rich area was macro‐dissected. For MDS, DNA was obtained from fresh or frozen bone marrow samples. As shown in Table 1, mutations in *DNMT3A* and *TET2* were shared by AITL, DLBCL, and MDS. In AITL cells, *IDH2* mutation was additionally observed. Meanwhile, *TP53* mutation was additionally present in DLBCL and MDS. The *TP53* mutation in the DLBCL sample presumably resulted in biallelic inactivation of the gene, because the mutation was accompanied by a deletion in the *TP53* locus, and the variant allele frequency (VAF) of the *TP53* mutation was 74%. In the bone marrow samples, *DNMT3A* and *TET2* mutations were detected at the time of the diagnosis of AITL, while cytogenetic analysis showed normal findings (Figure 2). After autologous transplantation, VAF of the *DNMT3A* and *TET2* mutations increased, and the TP53 mutation emerged; cytogenetic analysis showed the presence of monosomy 7. At the time of the occurrence of DLBCL, VAF of the *TP53* mutation increased in parallel with that of the *DNMT3A* and *TET2* mutations; cytogenetic analysis showed additional abnormalities including, deletion 5q and trisomy 8. Finally, VAF of the *TP53* mutation alone showed a steep increase up to 77%, indicating progression to biallelic targeting at the *TP53* locus in the MDS cells (Figure 2).

**TABLE 1:** Hematologic malignancies in three lineages that occurred in the patient

Diagnosis	AITL	DLBCL	MDS
**Clinical onset**	X	X + 31 months	X + 38 months
**Clinical manifestation**	Systemic lymphadenopathy	Perforation of the terminal ileum due to tumor cell infiltration	Prolonged cytopenia after chemotherapy for DLBCL
**Pathological findings**	Proliferation of medium‐sized clear cells, positive for CD3, CD4, and PD‐1	Diffuse proliferation of large atypical cells, positive for CD20, Bcl‐2, and MUM‐1	Trilineage dysplasia without an excess of blast
**Mutation analysis (VAF %)**			
** DNMT3A: p.W860R**	19.6	34.0	41.5
** TET2: p.D1143fs**	18.5	35.0	39.3
** TET2: p.G1218fs**	8.1	48.7	34.5
** TP53: p.R248W**	Not detected	74.0	59.8
** IDH2: p.R172G**	4.3	Not detected	Not detected

Abbreviations: AITL, angioimmunoblastic T‐cell lymphoma; DLBCL, diffuse large B‐cell lymphoma; MDS, myelodysplastic syndromes; VAF, variant allele frequency.

## 3 DISCUSSION

AITL cells frequently harbor mutations in genes encoding epigenetic modifiers, including *TET2* (76‐83%), *DNMT3A* (26‐38%), and *IDH2* (20‐45%) [3, 4, 5, 6, 7], and all of these mutations are also commonly observed in myeloid malignancies. Interestingly, an increasing number of the recent studies have revealed the clonal relationships between AITL and myeloid malignancies [8, 9, 10, 11]. Moreover, approximately 10% of AITL patients develop B‐cell malignancies during the clinical course of the disease [12]. Although B‐cell malignancies accompanying AITL are often positive for EBV, a substantial number of cases are negative for EBV, suggesting that factors other than EBV contribute to the lymphomagenesis [12]. To date, the clonal relationship between AITL and DLBCL remains unclear, although CD20(+) B‐cells infiltrating into AITL have been reported to have *TET2* and *DNMT3A* mutations in common [13].

Genomic profiling of this patient suggests that AITL arose from a founder clone harboring *DNMT3A* and *TET2* mutations; the addition of the *IDH2* mutation was presumably associated with progression to AITL. Sequential profiling additionally suggests that DLCBL and MDS arose from clones that had clonal evolution from a founder clone with the addition of the *TP53* mutation. Whereas *TET2* and *IDH2* mutations are mutually exclusive in myeloid malignancies [14], *TET2* and *IDH2* are often co‐mutated in AITL as observed in the present case [13]. *RHOA* mutations, which have been reported in 53%–71% of AITL patients [15, 16], were not detected in this case.

In addition, the kinetics of VAF in the bone marrow in the patient demonstrated the role of the *TP53* mutation in the progression of MDS. *TP53* mutation emerged in the bone marrow during the clinical course, and clones with *DNMT3A*, *TET2*, and *TP53* mutations expanded over a couple of years. The patient experienced rapid progression of MDS in the last stage, when the VAF of *TP53* showed a steep increase that suggested the occurrence of an additional hit, resulting in the biallelic targeting of *TP53*. A recent study has clearly demonstrated that multiple hits in the *TP53* gene had a remarkably negative impact on the outcome of MDS patients compared to a single hit [17].

In conclusion, we have described a patient with AITL who developed DLBCL and MDS subsequently. The hematologic malignancies in the three lineages, namely T‐cell, B‐cell, and myeloid lineages, had common mutations in the genes encoding epigenetic modifiers. The present findings strongly support the theory of multistep and multilineage tumorigenesis from a common founder clone with lineage‐specific additional events.

## CONFLICT OF INTEREST

The authors declare that there is no conflict of interest that could be perceived as prejudicing theimpartiality of the research reported.
